# Analysis of Tuberculosis Epidemiological Distribution Characteristics in Fujian Province, China, 2005-2021: Spatial-Temporal Analysis Study

**DOI:** 10.2196/49123

**Published:** 2024-11-18

**Authors:** Shanshan Yu, Meirong Zhan, Kangguo Li, Qiuping Chen, Qiao Liu, Laurent Gavotte, Roger Frutos, Tianmu Chen

**Affiliations:** 1 State Key Laboratory of Vaccines for Infectious Diseases, Xiangan Biomedicine Laboratory, School of Public Health, Xiamen University, No 4221-117, Xiang'an South Road, Xiang'an District, Xiamen City, 361101, China, 86 13661934715; 2 State Key Laboratory of Molecular Vaccinology and Molecular Diagnostics, National Innovation Platform for Industry-Education Integration in Vaccine Research, Xiamen University, Xiamen City, China; 3 Emergency Response and Outbreak Management Section, Fujian Provincial Center for Disease Control and Prevention, Fuzhou City, China; 4 Centre de Coopération Internationale en Recherche Agronomique (CIRAD), UMR 17 Intertryp, Montpellier, France; 5 Faculty of Medicine-Ramathibodi Hospital, Mahidol University, Bangkok, Thailand; 6 Espace-Dev, Université de Montpellier, Montpellier, France

**Keywords:** tuberculosis, TB, epidemiology, Fujian Province, temporal-spatial pattern, spatial-temporal analysis, surveillance, pulmonary tuberculosis, epidemic status, detection

## Abstract

**Background:**

Tuberculosis (TB) is a chronic infectious disease that harms human health for a long time. TB epidemiological distribution analysis can help governments to control TB in high TB incidence areas. The distribution trend of TB cases varies in different regions. The unbalanced temporal and spatial trends of pulmonary TB (PTB) risk at a fine level in Fujian Province remain unclear.

**Objective:**

The purpose was to analyze different distribution characteristics, explore the prevalence of TB in this region, and provide a scientific basis for further guidance of TB control work in Fujian Province, China.

**Methods:**

Prefectural-level and county-level notified PTB case data were collected in Fujian Province. A joinpoint regression model was constructed to analyze the unbalanced temporal patterns of PTB notification rates from 2005 to 2021 at prefecture-level city scales. The spatial clustering analysis and spatial autocorrelation analysis were performed to assess the inequality of the locations of PTB cases. Demographical characteristics were explored by the method of descriptive analysis.

**Results:**

TB cases reported in Fujian showed an overall downward trend from 2005 to 2021 (in 2005: n=32,728 and in 2021: n=15,155). TB case numbers showed obvious seasonal changes. The majority of TB cases were middle-aged and older adult male patients (45 years and older; n=150,201, 42.6%). Most of the TB cases were farmers (n=166,186, 47.1%), followed by houseworkers and the unemployed (n=48,828, 13.8%) and workers (n=34,482, 9.8%). Etiologically positive TB cases continue to be the main source of TB cases (n=159,702, 45.3%). Spatially, the reported TB cases were mainly distributed in cities in southeastern Fujian, especially at the county level. TB case numbers showed 2 spatial groups; cases within each group shared similar case characteristics. In terms of geographical distribution, TB showed obvious spatial correlation, and local areas showed high aggregation.

**Conclusions:**

The TB incidence trend decreased annually in Fujian Province. TB cases distributed commonly in the male population, middle-aged and older people, and farmers. Etiologically positive cases are still the main source of *Mycobacterium tuberculosis* infection. TB incidence is higher in the cities with a developed economy and large population in the southeast. TB control should be strengthened in these populations and areas, such as via early screening of cases and management of confirmed cases.

## Introduction

Tuberculosis (TB), a chronic infectious disease, has been endangering human health over the years. In Europe, in the 17th and 18th centuries, TB was known as the “white plague,” infecting almost 100% of the population and killing 25% of the population [1,2]. As one of the high-burden countries, Chinese TB control still needs to be strengthened [3]. Over the years, TB incidence has shown a downward trend year by year. The number of people with multidrug-resistant TB is steadily increasing worldwide, reaching about 450,000 in 2021 [4,5]. Globally, only 57% of patients with multidrug-resistant TB have been successfully treated [3]. Fujian Province, located in the southeast of China, is one of the country’s high-income provinces. The Fujian provincial government has made efforts to gradually establish a TB health service system and implement prevention and control strategies [6], but there is still a gap from the overall goal of ending TB. Drug-resistant strain diversity and drug-resistant TB incidence also have a great impact on the effectiveness of TB control in this region [7,8]. Therefore, it is urgent to have a deep understanding of the distribution and epidemiological characteristics of TB and concentrate limited health resources to control the spread of TB in core areas, which will be very beneficial to the TB control work in this region.

Spatial and temporal analyses of urban TB and its driving mechanism are helpful to reveal the spatial distribution pattern, transmission mechanism, and evolution mechanism of the epidemic. These analyses are commonly used to study the epidemiological characteristics of TB, such as temporal descriptive analysis, spatial-temporal analysis, and so on. They have a powerful function in characterizing the epidemic of infectious diseases. Previous studies have used the joinpoint regression (JPR) model [9], Moran *I* spatial autocorrelation analysis [10], spatial scan statistical method [11], Bayesian model [12,13], and so on. Many studies have analyzed the temporal patterns and trends of TB cases using the JPR model [9,14,15]. Meanwhile, spatial autocorrelation analysis has been popularly used to explore the spatial-temporal heterogeneity of pulmonary TB (PTB) in recent years [10,13,14,16-25]. However, only 1 study examined the status of TB in Fujian Province through global and local spatial autocorrelation analysis, visual hot spot analysis, and spatial-temporal scanning analysis [26,27]. Previous studies have broadened the understanding of the spatial and temporal pattern of TB in Fujian Province, but there are some limitations due to the lack of long-term spatial and temporal pattern evidence. In this study, in addition to exploring its influencing factors from the perspective of overall TB, we also focused on the distribution characteristics of etiologically positive and etiologically negative TB cases.

Therefore, this study further explored the distribution characteristics of *Mycobacterium tuberculosis* (MTB), providing more effective scientific help for the further control of TB in Fujian Province, as well as the method basis for scientific control of TB in other cities in China, and jointly helping China achieve the goal of ending TB as soon as possible.

## Methods

### Data Collection

In this study, the epidemiological data of notified cases of PTB from 2005 to 2021 were analyzed. The data were sourced from the Fujian Provincial Center for Disease Control and Prevention and included 9 prefecture-level regions (including Fuzhou, Xiamen, Quanzhou, Zhangzhou, Putian, Longyan, Sanming, Nanping, and Ningde prefecture-level cities) and 84 county-level regions (including 31 districts, 11 county-level cities, and 42 counties; Figure S1 in Multimedia Appendix 1). The data collected for each case included demographic information, such as age, gender, occupation, address, and anti-TB drug history, as well as diagnostic information, including date of diagnosis, date of onset, and diagnostic result. Population data for each county at year-end were obtained from the yearbook of the Fujian Provincial Bureau of Statistics. The annual notification rate of PTB in a region was calculated as the ratio of the annual number of notified PTB cases to the total population at the end of the year in the region.

### Classification of PTB

The diagnosis of active TB is based on a combination of clinical symptoms, radiographic findings, sputum smear examination, and culture results. In accordance with the National Notifiable Diseases Surveillance System guidelines, published in 2018, cases of TB are classified into 4 categories: “pathogen-positive” (including both sputum smear–positive and culture-positive cases of PTB), “pathogen-negative” (including sputum smear–negative cases of PTB), “rifampicin-resistant,” and “no pathogenic findings” (including cases of extrapulmonary TB and tuberculous pleurisy) [28]. For the purposes of this study, all historical data were reclassified according to the latest National Notifiable Diseases Surveillance System guidelines in order to ensure consistency in the data.

### Statistical Methods

Three methods were used to analyze TB epidemiology characteristics (see details in Multimedia Appendix 2).

First, JPR models were used to calculate the TB temporal trends, which are often used to describe changes in trends. The annual percentage change (APC) for TB incidence rates was used to quantify the time trends, and it also estimates the average APC in the whole period studied [29,30].

Second, the spatial autocorrelation index is an important index to measure the potential interconnectedness and dependence of study objects in a region. The Moran index (Moran *I*) is a statistical method used to measure spatial autocorrelation, which can help analyze clustered or discrete patterns in geographic data or other datasets with spatial properties. It is divided into global Moran *I* (*I*) and local Moran *I* (*I_i_*). The Getis-Ord Gi* statistic is used to identify the statistically significant hot and cold spots.

Finally, cluster analysis is used to further explore the internal relationships of different clusters. Unsupervised hierarchical cluster analysis was conducted to identify relevant groups of patients with TB based on 9 prefecture-level cities and 83 county-level cities. The analysis was performed using the R statistical software package (R Foundation for Statistical Computing) with a Euclidean distance measure and Ward linkage. In addition, statistical analyses for association and correlation were conducted using a variety of methods including the Fisher exact test, ANOVA, chi-square test, and the independent sample *t* test (2-tailed) to further investigate the relationship between the identified clusters and relevant demographic and clinical characteristics of the patient population.

### Ethical Considerations

The data were obtained from the Chinese Information System for Diseases Control and Prevention. This effort of investigation was part of the Fujian Provincial Center for Disease Control and Prevention’s routine responsibility; therefore, institutional review and informed consent were not required for this study on the following grounds: (1) only anonymized records were used without the need for direct involvement nor active participation of patients, (2) neither medical intervention nor biological samples were involved, and (3) study procedures and results would not affect clinical management of patients in any form.

## Results

**Figure 1.**
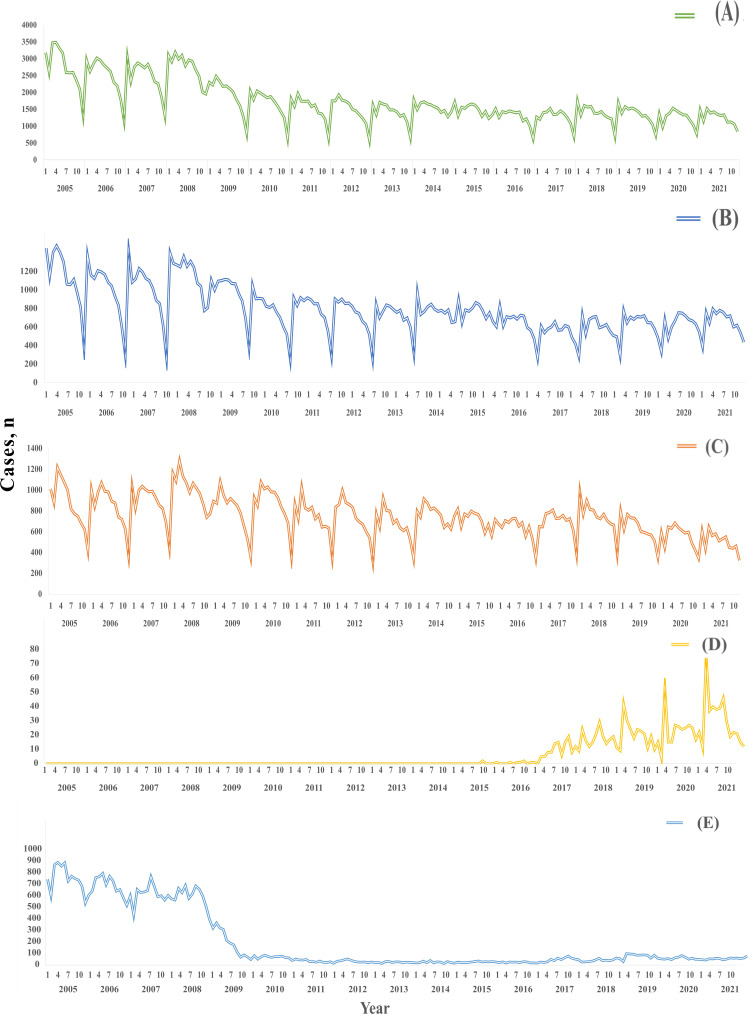
Temporal trend of PTB in Fujian Province, 2005‐2021. (**A**) All PTB, (B) pathogen-positive PTB, (**C**) pathogen-negative PTB, (**D**) rifampicin-resistant PTB, and (**E**) no pathogenic findings PTB. PTB: pulmonary tuberculosis.

TB cases reported in Fujian showed an overall downward trend from 2005 to 2021 (in 2005: n=32,728 and in 2021: n=15,155). The downward trend was also observed in pathogen-positive, pathogen-negative, and no pathogenic findings PTB cases but not in rifampicin-resistant PTB cases (Figures1  2). The incidence was the highest in Ningde city. It was the city with the largest decline in TB incidence from 2005 to 2012 (in 2005: 130.36 per 100,000 and in 2012: 54.61 per 100,000). The incidence rates showed a relatively stable decreasing trend in Fuzhou, Longyan, Quanzhou, and Sanming cities. In contrast, the incidence rate in Putian city showed a fluctuating trend, increasing from 60.25/100,000 in 2005 to 71.06/100,000 in 2008, and subsequently declining to 38.49/100,000 in 2021 (Multimedia Appendix 3).

TB case numbers showed obvious seasonal changes. The 2 high peaks were distributed in March and September, and the low peak was in December. This seasonal pattern was also observed in the distribution of pathogen-positive TB and pathogen-negative TB cases.

The majority of TB cases were middle-aged and older adult male patients (45 years and older; n=150,201, 42.6%). There were more male cases than female cases (male: n=258,225, 73.2% and female: n=94,684, 26.8%; sex ratio 2.73; *P*<.001; Multimedia Appendices 4 and 5).

Most of the TB cases were farmers (n=166,186, 47.1%), followed by houseworkers and the unemployed (n=48,828, 13.8%) and workers (n=34,482, 9.8%). These 3 occupational groups account for more than 70% (249,496/352,909) of all TB cases in Fujian Province.

Etiologically positive TB cases continue to be the main source of TB cases. They accounted for 45.3% (n=159,702) of TB cases (n=152,256, 43.1% of etiologically negative TB cases; Figure 3). There were fewer cases of TB in the other 2 diagnosed types (no pathogenic cases detected: n=39,689, 11.2% and rifampicin-resistant cases: n=1262, 0.4%). In addition, we observed internal heterogeneity in the distribution of patients with pathogen-positive TB or pathogen-negative TB among prefecture-level cities, such as Sanming city, with 4 different colors. The number of drug-resistant TB cases has been on the rise since 2016, with patients who are rifampicin-resistant concentrated in Ningde and Putian, indicating higher prevalence in these regions. About 0.4% (n=1262) of cases were rifampicin-resistant. This is due to the diagnostic classification resulting from the adjustment of the TB reporting classification criteria in 2018. Because the data are inconsistent and the proportion is relatively small (<5%), this part of the data is discarded for analysis.

**Figure 2.**
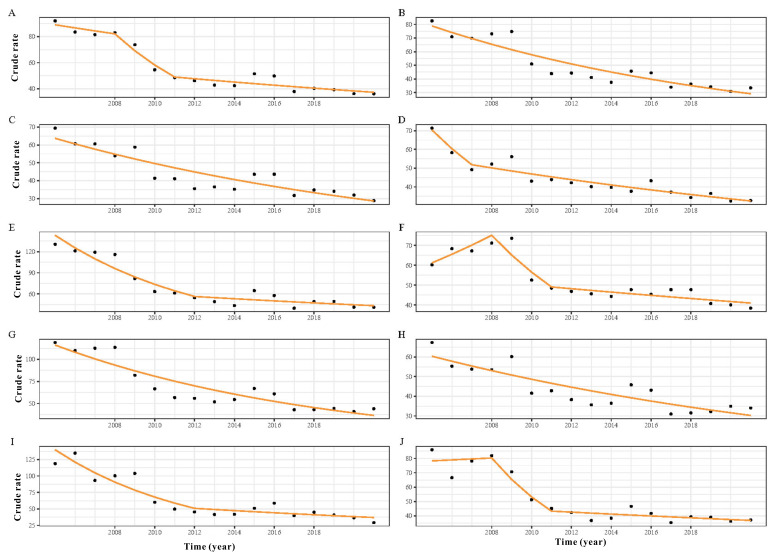
Trend in crude incidence rate of the whole province and 9 prefecture-level cities according to the joinpoints identified by the analysis. Solid lines result from a logistic regression fitting a joinpoint regression model as the outcome variable. Real crude rate (dots) and fitted crude rate (lines) data are shown for each prefecture-level city—(A) Fujian Province, (B) Fuzhou, (C) Longyan, (D) Nanping, (E) Ningde, (F) Putian, (G) Quanzhou, (H) Sanming, (I) Xiamen, and (J) Zhangzhou—and year.

**Figure 3.**
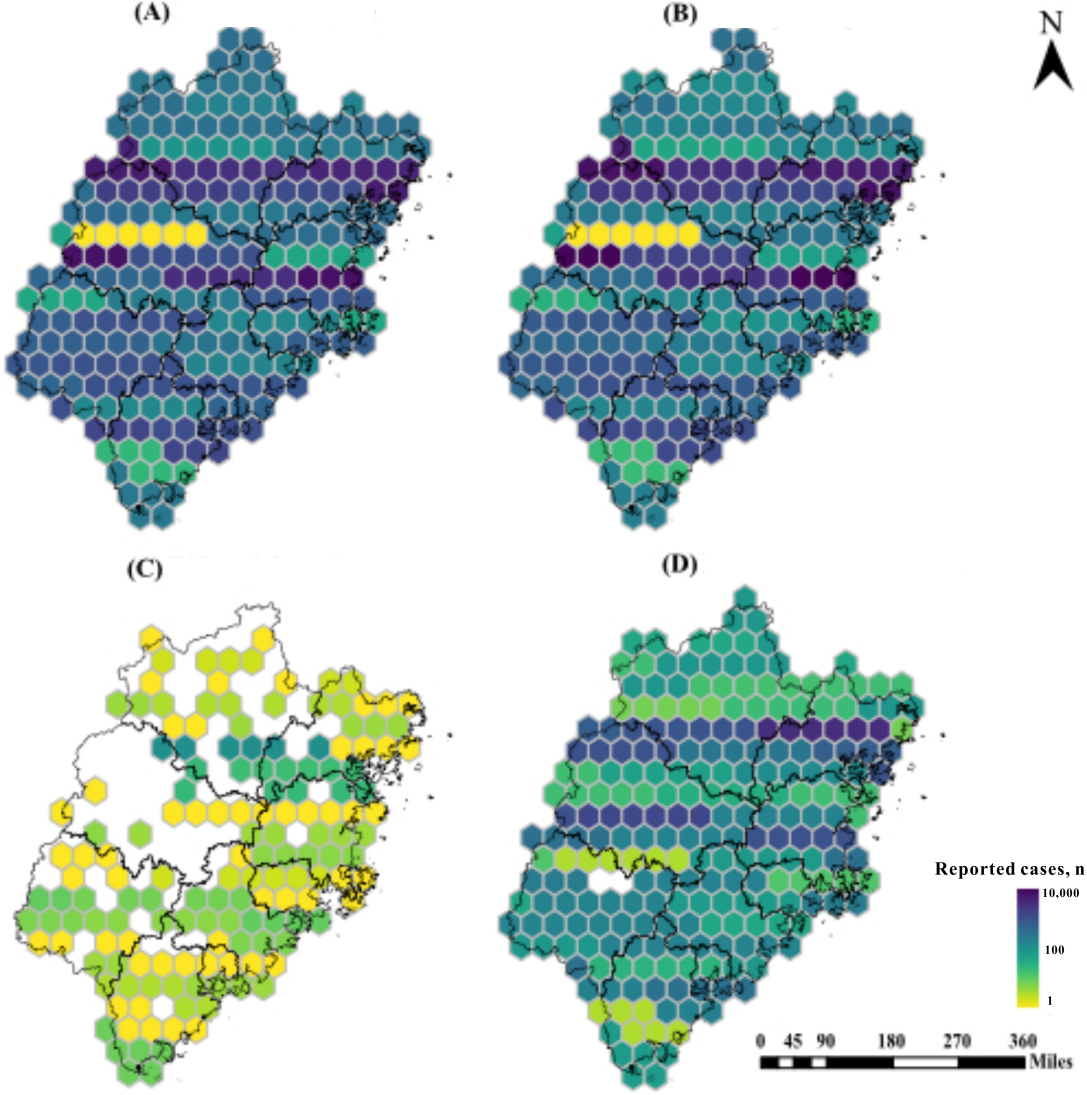
Choropleth maps of notification number of PTB cases in Fujian Province from 2005 to 2021 in Hexagon Commonwealth based on smear status. (A) Pathogen-positive PTB, (B) pathogen-negative PTB, (C) rifampicin-resistant PTB, and (D) no pathogenic findings. PTB: pulmonary tuberculosis.

**Figure 4.**
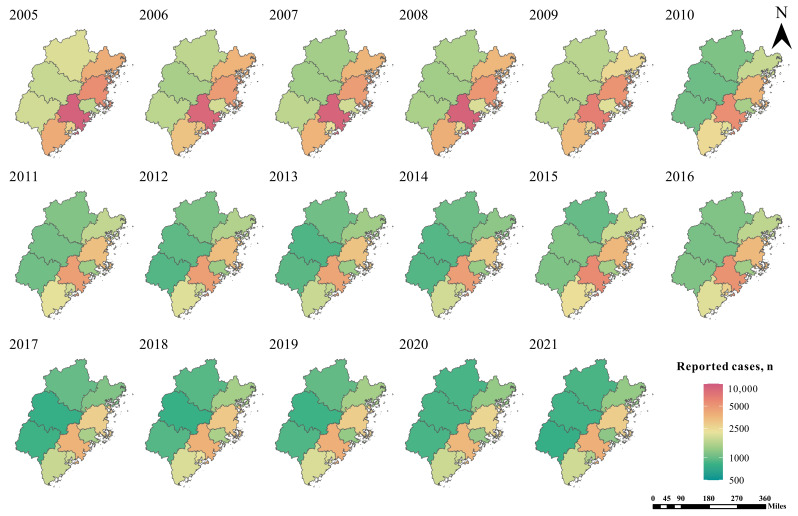
The geographical distribution of annualized average incidence of pulmonary tuberculosis in Fujian Province from 2005 to 2021.

Spatially, the reported TB cases were mainly distributed in cities in southeastern Fujian, especially at the county level (Figure 4). At the municipal level, the number of TB cases was mainly concentrated in Quanzhou (n=94,860, 26.9%), Fuzhou (n=62,328, 17.7%), and Zhangzhou (n=42,606, 12.1%), and Sanming (n=18,942, 5.4%) had the least number of cases. At the county level, the counties with the highest average number of TB cases were Jinjiang, Nan’an, and Hui’an in Quanzhou city and Fu’an in Ningde city (Figures S2-S5 in Multimedia Appendix 1). It is worth noting that Quanzhou had the highest average annual incidence, and all reported rifampicin-resistant cases were from Quanzhou city. In addition, the comparison of the coefficient of variation between cities showed that the spatial heterogeneity of the infection rate of 100 km^2^ was the strongest, the spatial heterogeneity of the infection rate of 1 million km^2^ was the weakest, and the number of patients was in the middle. The coefficient of variation of the 3 indexes is greater than 7, much greater than 0.36, and the degree of difference is high (Multimedia Appendix 6).

**Figure 5.**
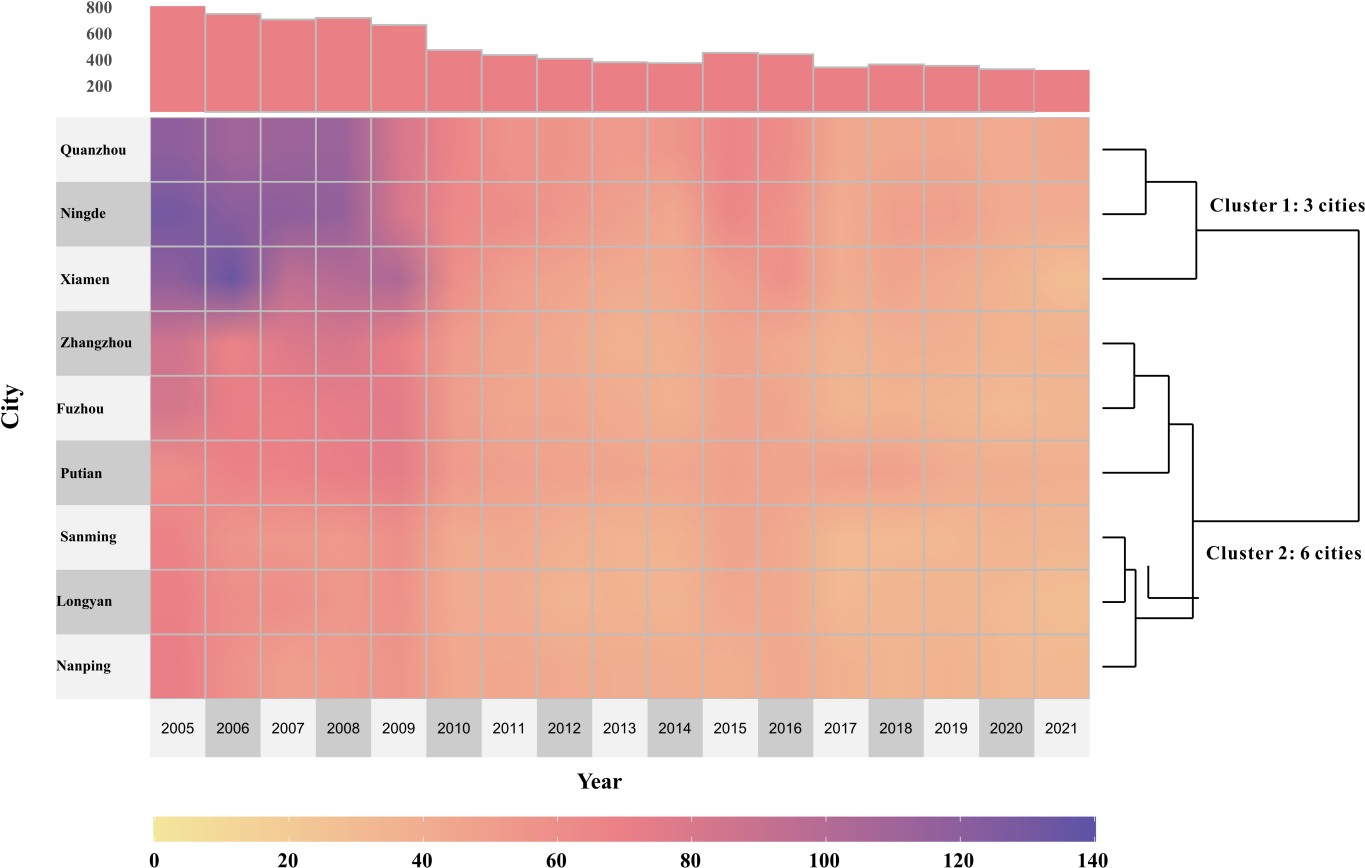
Hierarchical clustering analysis of the regions in Fujian Province based on the incidence of PTB from 2005 to 2021. Each column of the heat maps represents 1 year, and each row represents 1 region. Both the years and the regions have been clustered based on the similarity of their incidence of PTB. The color of the cells indicates the annual PTB incidence of each region for the corresponding year. The main enriched pathways related to each region cluster are included. PTB: pulmonary tuberculosis.

TB case numbers showed 2 spatial groups; cases within each group shared similar case characteristics. Using an unsupervised hierarchical clustering method (Figure 5), 3 cities were classified into category 1 (Quanzhou, Ningde, and Xiamen) and 6 cities into category 2 (Zhangzhou, Putian, Fuzhou, Sanming, Longyan, and Nanping) based on the similarity in the number of annual reported TB cases. Subsequently, a detailed cluster analysis was performed for each cluster, especially for 84 county-level cities. The analysis reveals 2 distinct subgroups in cluster 1, called cluster 1-A and cluster 1-B (comprising 6 and 20 counties, respectively; Figure S6 in Multimedia Appendix 1). Similarly, 3 distinct subgroups are found in cluster 2, known as cluster 2-A, cluster 2-B, and cluster 2-C (comprising 35, 1, and 21 counties, respectively; Figure S7 in Multimedia Appendix 1).

**Figure 6.**
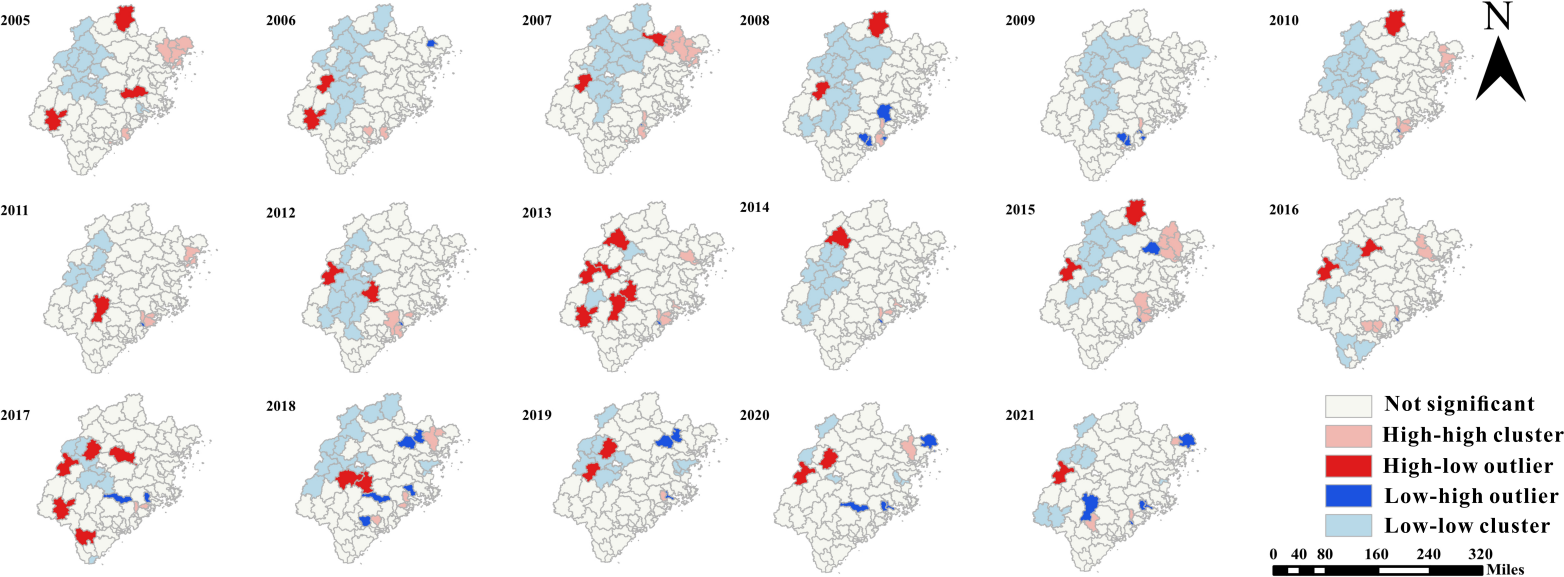
Spatial clustering of pulmonary tuberculosis incidence at the county level in Fujian Province, 2005-2021, based on local indicators of spatial association using Anselin local Moran *I* statistic. Only those counties whose local Moran *I* has reached the significance level of .05 are present on the map.

**Figure 7.**
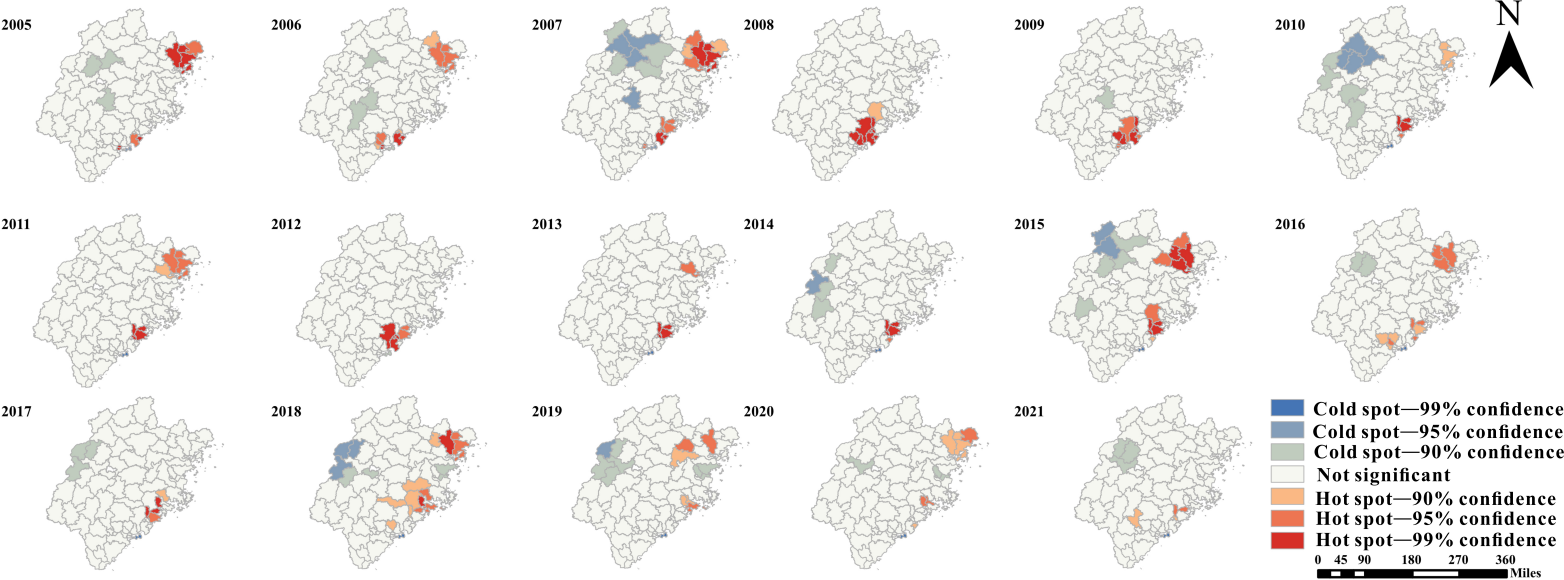
Spatial clustering of pulmonary tuberculosis incidence at the county level in Fujian Province, 2005-2021, based on the Getis-Ord Gi* statistic. Only those counties whose local Moran *I* has reached the significance level of .05 are present on the map.

In terms of geographical distribution, TB showed obvious spatial correlation, and local areas showed high aggregation. The global spatial autocorrelation analysis of PTB showed that there was a significant cluster distribution of PTB since 2005. Annual global Moran *I* reached a significance level of .05 (Multimedia Appendix 7). With the exception of 2017, all global Moran *I* values are greater than 0 at 5%. The spatial pattern is clustered in most years, with high clustering occurring in recent years (2008, 2013‐2015, 2017‐2018, and 2020‐2021; Multimedia Appendix 8). To further understand the geographical distribution characteristics of urban TB risk, local Moran *I* was used to analyze the 4 types of clusters: high-high, high-low, low-low, and low-high. In terms of infection risk, the spatial heterogeneity pattern of high-high and low-low remained stable in the whole area, but high-low and low-high showed large variation and difference in the south. During the reporting year, the detected PTB high-high clusters were steadily distributed in eastern Fujian Province, mainly concentrated in the county area of Ningde city (Figure 6). The results are basically consistent with the above results of geographic descriptive analysis. At the same time, the high-high cluster was formed in Quanzhou city, in the south of Fujian Province. The results also show that the low-low cluster was consistently located in different counties over the reported years, excluding the north and west. Every year, low-high clusters are distributed in Ningde and Putian cities. The high-low group disappeared in 2009 and appeared in other years, and its location was randomly distributed. The results of the Getis-Ord Gi* analysis showed that the cold and hot spots of TB incidence were shown in Figure 6 from 2005 to 2021, with confidence of 90%, 95%, and 99%, respectively. TB incidence showed an obvious downward trend and gradually increased. The hot spots of PTB were located in Ningde (eastern) and Quanzhou (southern) cities, and it gradually showed hot spots in Putian city. The central counties showed a trend of high-value agglomeration. In recent years, high-value agglomeration centers had moved from east to south. The cold spot was mainly located in the north, with some areas in the central or eastern part of the province (Figure 7).

## Discussion

### Principal Findings

This paper describes the epidemiological distribution of TB cases in Fujian Province in the past 16 years by using different methods, including time distribution, population distribution, and spatial distribution. TB incidence in Fujian Province showed a downward trend, and the cases were mostly among the male population, middle-aged and older people, and farmers. The majority of TB cases were etiologically positive cases, and the spatial distribution of TB cases was mainly in the densely populated southeastern cities.

TB case numbers in Fujian Province showed an overall downward trend with seasonality, which was consistent with the national TB trend. There were obvious regional differences in the PTB notification rate and APC for PTB. The APC ranges from −15.8 to −2.7, which is better than the national overall reduction of 4.2% [9]. The decline in TB incidence can be attributed to the rising economic level, improved social conditions, effective treatment, and public health interventions [31]. In addition, the seasonal trend illustrated that most cases were observed during the spring and autumn, with the trough in December. This finding fits well with the previous studies [32-34]. Some reasons can explain this. First, it is possible that the transmission pattern of MTB is inherently seasonal, which is still controversial [35-37]. Second, it may be related to Chinese customs, such as the Chinese Lunar New Year. At such moments, Chinese people are reluctant to seek treatment for illnesses unless they are serious. Therefore, some cases with less serious symptoms will choose to go to the hospital for treatment after the New Year, that is, spring. This is one of the reasons for the high incidence in spring and the low incidence in winter. In addition, the spring and autumn of each year is also the season of enrollment and entry examination. There will also be some cases detected by physical examination. Finally, seasonal changes and high rates of respiratory disease may promote the spread of MTB and accelerate TB development [38]. Common respiratory diseases, such as influenza, swine flu, mycoplasma pneumonia, and other diseases, are high in winter and spring. These diseases tend to reduce human immunity. Coupled with seasonal changes, MTB is easy to break through the human immune barrier in the human body and continue to spread. Due to the slow progression of TB, these cases tend to develop into confirmed TB cases in spring and autumn [35,39]. This may also be one reason for the seasonal peaks.

Male populations, farmers, and middle-aged and older adults are among the most prevalent TB populations in Fujian Province. Male populations were at risk of TB compared to female populations. One study found that TB prevalence is significantly higher among the male population than among the female population, with strong evidence that the male population is disadvantaged in seeking or accessing TB care in many settings [40]. Another study found distinct mixing patterns by sex and TB disease status, including that TB cases have proportionally more adult male contacts and fewer contacts with children [41]. Older people and adults were more prone to be infected than younger people. Older age groups bear a higher risk of TB progression due to a weakening immune system. As life expectancy increases along with population aging, these age disparities in TB burden may increase the need to prioritize TB control measures among older people [31]. Farm workers (farmers) are approximately 5 times more likely to develop TB than the general population of employed workers [42,43]. Urban population accounts for 71.04% of the province’s total population in 2023 [44]. Farmers accounted for about 30% of the population but became the highest TB incidence group, indicating that literacy and economic ability are very important for TB control. Low education levels prevent farmers from correctly understanding TB. As a result, they could not get treatment timely when symptoms first appear [45,46]. In addition, low income is also one of the reasons for their treatment [47]. The knowledge of TB prevention and control should be strengthened in farmers, and the screening rate of TB should be improved to detect and treat patients with TB as early as possible.

Pathogen-positive TB cases remain the main TB infectious source, and pathogen-positive TB is strongly infectious and greatly harmful to the human body. According to the Statistical Yearbook of Fujian Province in 2023, the top 5 cities in terms of the province’s health resources are Fuzhou, Quanzhou, Zhangzhou, Xiamen, and Longyan. These 5 cities occupy more than 70% of the province’s health resources in terms of the number of beds in health institutions and the allocation of health technicians (including licensed physicians and registered nurses) [44]. These cities are located in the southeastern part of Fujian Province and are also the cities with a high population and high TB case concentration. Control measures can be appropriately strengthened in these cities, such as the shortening of diagnosis delay [48], isolation of positive cases, timely treatment [49-51], and behavior correction [48,50]. In addition, the statistical analysis revealed the intercity heterogeneity of PTB notification rates based on smear status. It was found that the distribution of diagnosis types was not the same between cities. Corresponding measures can be taken respectively based on the various distribution characteristics. For example, screening of drug-resistant strains can be carried out accordingly, and the management of drug-resistant cases can be improved in high drug-resistance rate cities, such as Quanzhou, Ningde, and Putian. It is worth noting that Putian has the fifth largest population in the province, but its health resources rank at the bottom of the province (number of beds and number of health technicians). About 1.5 million Putian residents live or work abroad. They have a long stay outside of China in 85 countries and regions but return to Putian city frequently. The city also has a high incidence of TB. TB control measures should pay special attention to screening of returnees and TB transmission factors due to population movements. In the diagnostic classification, 11.2% (n=39,689) of the cases were still those with no MTB detected, but anti-TB treatment was effective.

TB control effectiveness should be further strengthened in areas with a high incidence. The climate in Fujian is a subtropical climate, which is warm, humid, and not conducive for MTB survival [52]. There is a distinct difference in climate between north and south, coastal and inland regions, and valleys and mountains. Both annual temperature and precipitation increased from northwest to southeast. However, the notification rate in the eastern and southern regions was significantly higher than those in the northern, western, and central regions (*P*<.001). PTB spatial distribution incidence was characterized by multiple hot spots located in the east and south and cold spots in the northern areas. This may be due to the unbalanced economic levels leading to unbalanced treatment levels and medical systems [52]. The southeast Fujian area, which comprises 5 cities and counties (Fuzhou, Xiamen, Putian, Quanzhou, and Zhangzhou), is the economic hub of Fujian, accounting for 77% of the province’s gross domestic product in 2020 (US $ 616,442,163,732) [53]. In addition, these 5 cities account for 74% of the province’s population [44]. The density of the population determines that these 5 cities need to implement more stringent TB control efforts.

### Limitations

It is plausible that several limitations could have influenced the results obtained. First, the data used in this paper are based on official statistics; however, there is a possibility that some TB cases may have been omitted from the data due to the presence of underreport of TB cases, which means cases recorded in the internal hospital records were not entered into the national TB reporting systems. One study found that 26% of TB cases were unreported in selected hospitals in eastern China [54]. The indicators associated with underreporting of TB were children; migrants; patients with drug-resistant TB [55]; and smear status, whereby smear-negative cases are strongly related to underreporting and smear-positive cases are not quite related [56]. Underreporting of TB cases is possible for all regions, so their impacts on our analysis were not negligible.

Data from population-level surveys for undiagnosed TB carried out in several countries during the last 2 decades can be combined with data on notified cases to provide complete insight into the magnitude and nature of differences in TB. Second, the Moran test can only identify clusters of PTB at a specific time point but cannot quantify the spatial variation in temporal trends [57]. To further our research, we intend to explore the role of secular trends in effective contact for infectious cases. Additionally, it would be beneficial to incorporate more granular data, such as molecular data, in future research to understand further the proportion of TB transmission events caused by patients with pathogen-negative TB in Fujian Province.

### Conclusions

The TB incidence trend decreased annually in Fujian Province. TB cases distributed commonly in the male population, middle-aged and older people, and farmers. Etiologically positive cases are still the main source of MTB infection. TB incidence is higher in the cities with a developed economy and large population in the southeast. TB control should be strengthened in these populations and areas, such as via early screening of cases and management of confirmed cases.

## Supplementary material

10.2196/49123 Multimedia Appendix 1 Geographical distribution of pulmonary tuberculosis in different diagnostic categories and population cluster analysis at county level in Fujian Province.

10.2196/49123 Multimedia Appendix 2 Statistical methods.

10.2196/49123 Multimedia Appendix 3 Joinpoint analysis of newly notified tuberculosis cases in different cities of Fujian Province.

10.2196/49123 Multimedia Appendix 4 Demographic characteristics of pulmonary tuberculosis cases in Fujian Province based on number of annual reported cases, 2005-2021.

10.2196/49123 Multimedia Appendix 5 Demographic characteristics of pulmonary tuberculosis cases in Fujian Province from 2005 to 2021 based on 4 types of diagnosis results.

10.2196/49123 Multimedia Appendix 6 Spatial heterogeneity analysis of tuberculosis in Fujian Province cities.

10.2196/49123 Multimedia Appendix 7 The results of global spatial autocorrelation analysis in Fujian Province, 2005-2021.

10.2196/49123 Multimedia Appendix 8 The results of Getis-Ord General G analysis in Fujian Province, 2005-2021.

## Abbreviations

APC: annual percentage change
JPR: joinpoint regression
MTB: *Mycobacterium tuberculosis*
PTB: pulmonary tuberculosis
TB: tuberculosis
