# Supplementary material for: Analysis of Tuberculosis Epidemiological Distribution Characteristics in Fujian Province, China, 2005-2021: Spatial-Temporal Analysis Study
Source: JMIR Public Health Surveill. 2024 Nov 18;10:e49123. doi: 10.2196/49123 (PMC11590169; doi:10.2196/49123)
Supplement: Multimedia Appendix 1 [file publichealth-v10-e49123-s001.docx]

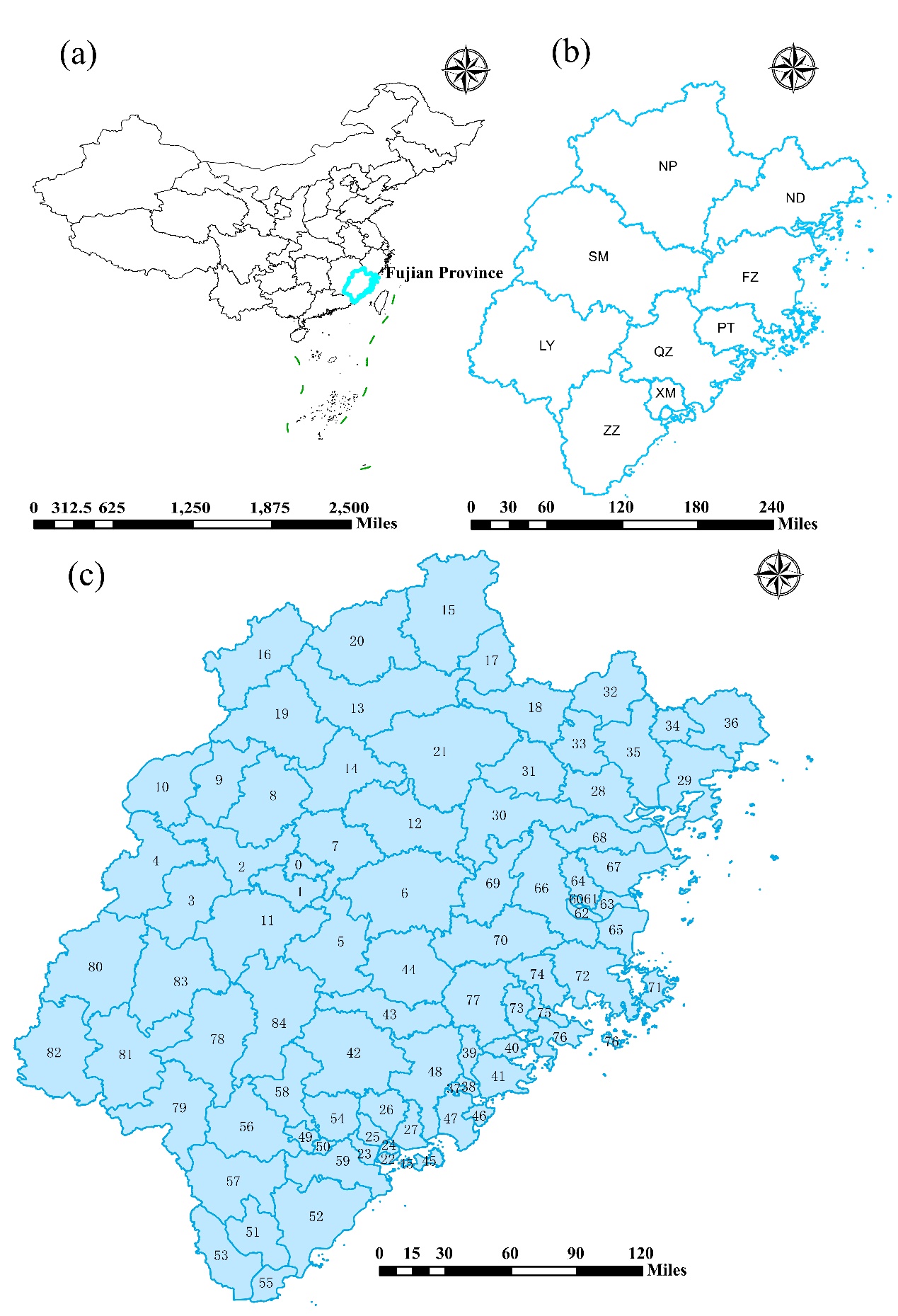


**Figure S1.** Location of study areas. **A) The geographical location of Fujian in China; B) The geographical location of 9 prefectures in Fujian; C) The geographical location of 84 counties in 9 prefectures.**

Fundamental geographic data were downloaded from the National Geomatics Centre of China to make the county-level polygon map of Fujian, and all cases were geocoded and matched to the map.


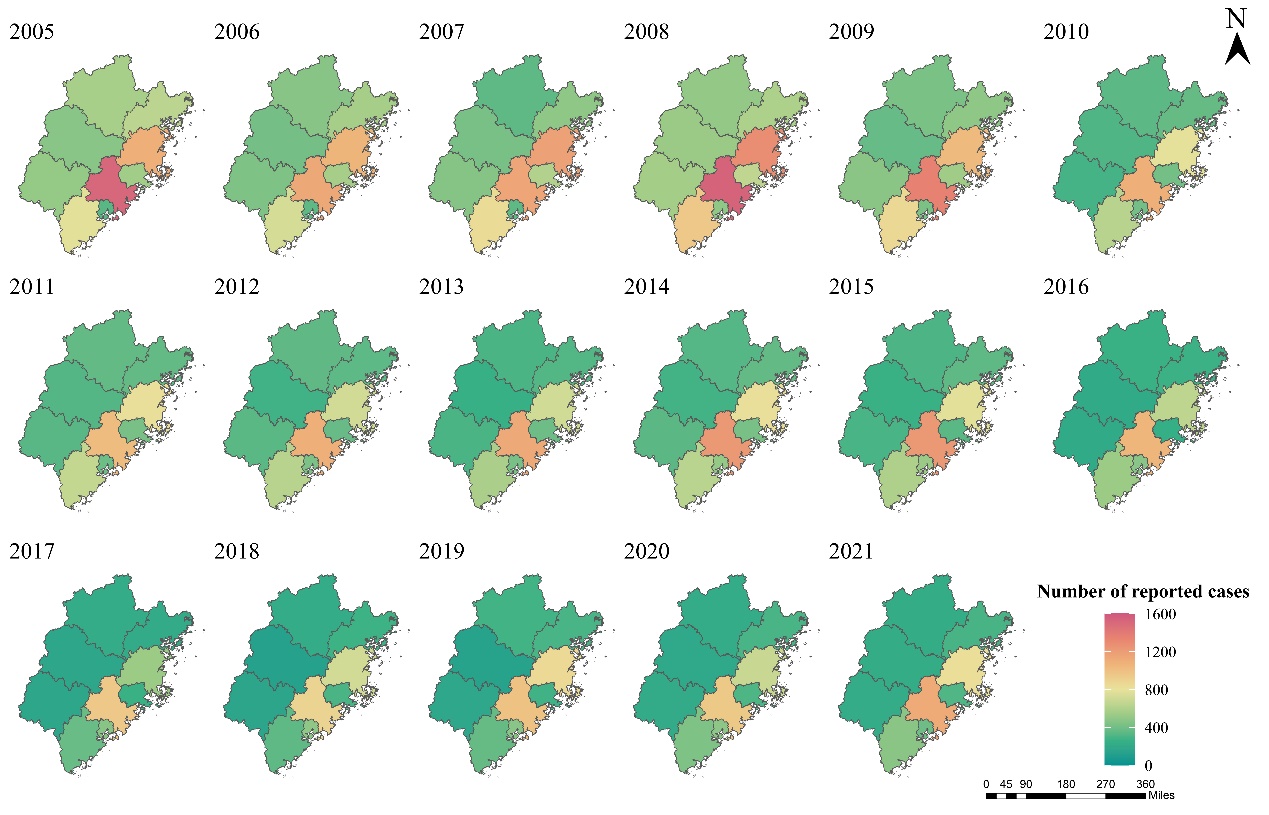


**Figure S2.** The geographical distribution of annualized average incidence of pathogen positive PTB in Fujian Province from 2005 to 2021.


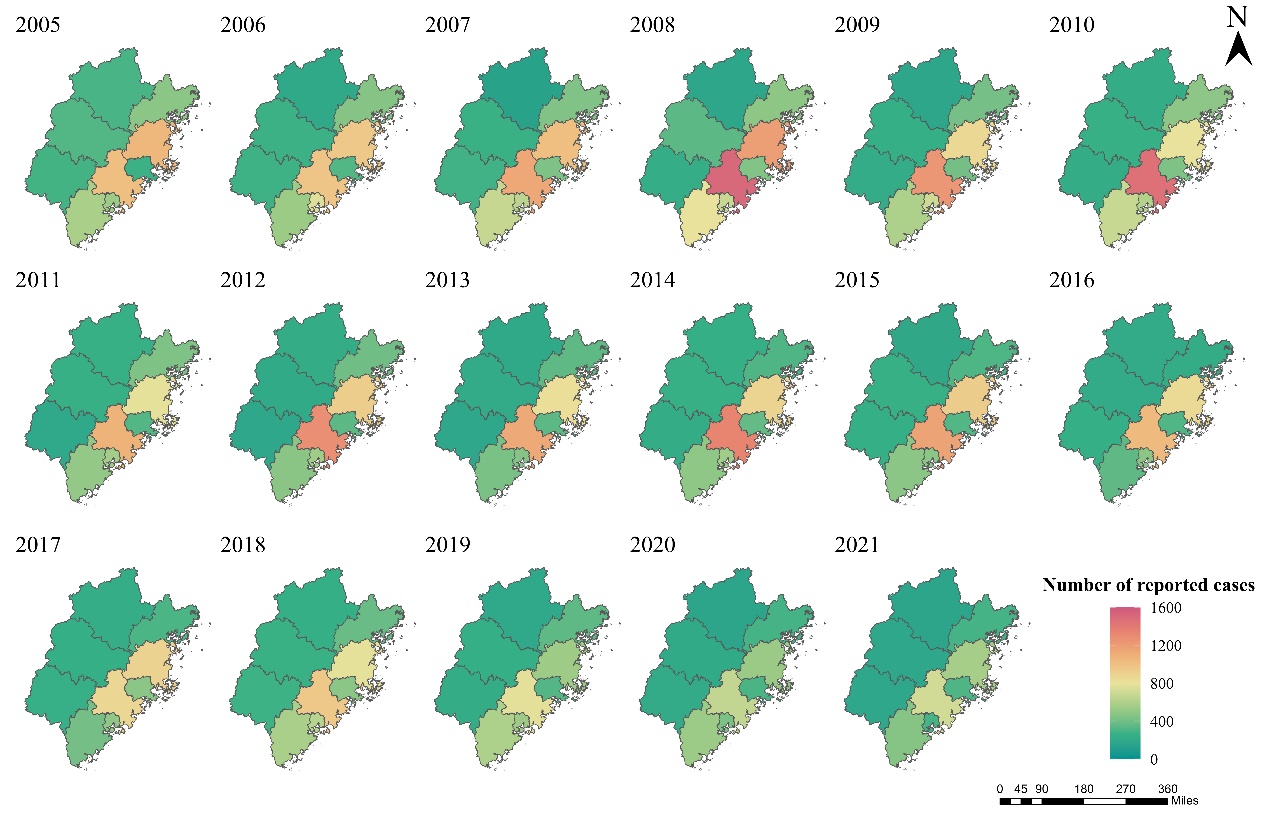


**Figure S3.** The geographical distribution of annualized average incidence of pathogen negative PTB in Fujian Province from 2005 to 2021.


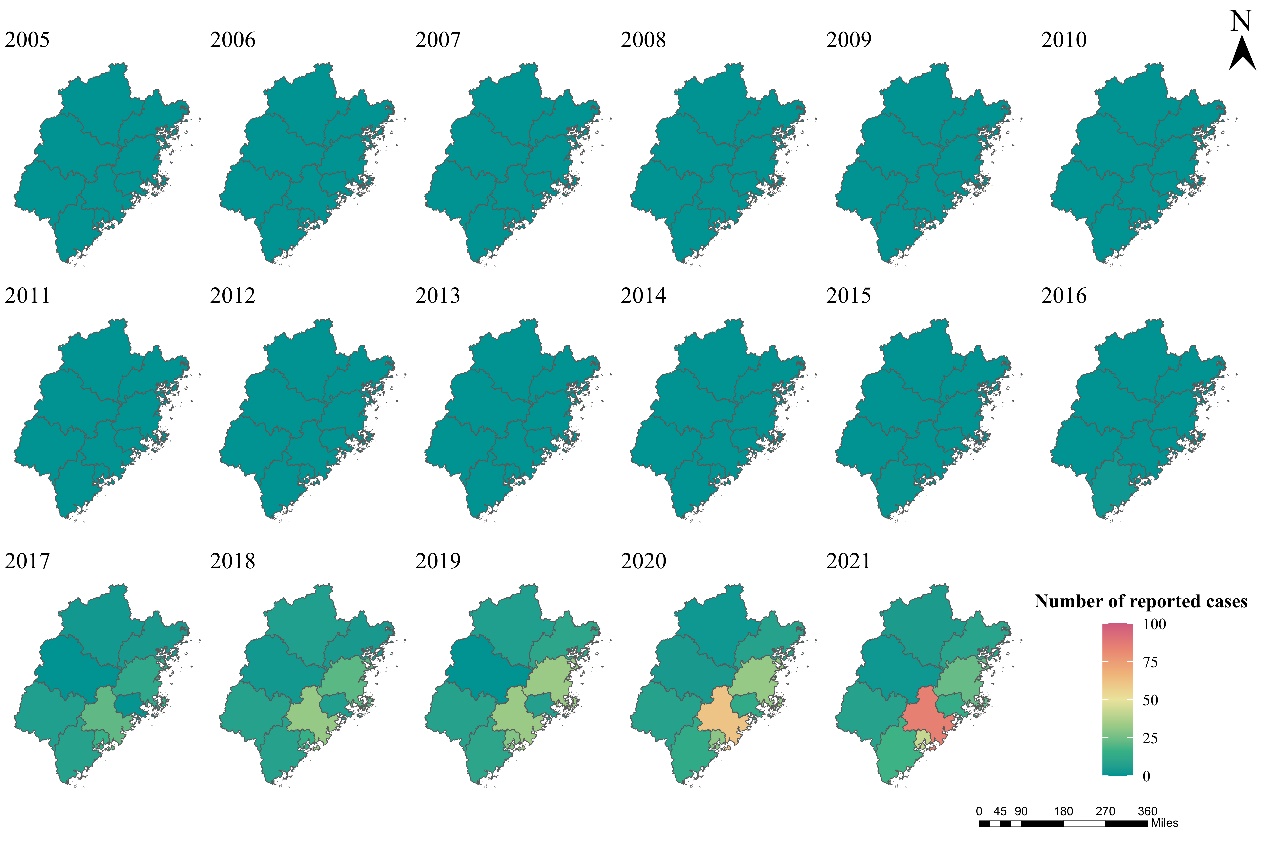


**Figure S4.** The geographical distribution of annualized average incidence of Rifampicin resistant PTB in Fujian Province from 2005 to 2021.


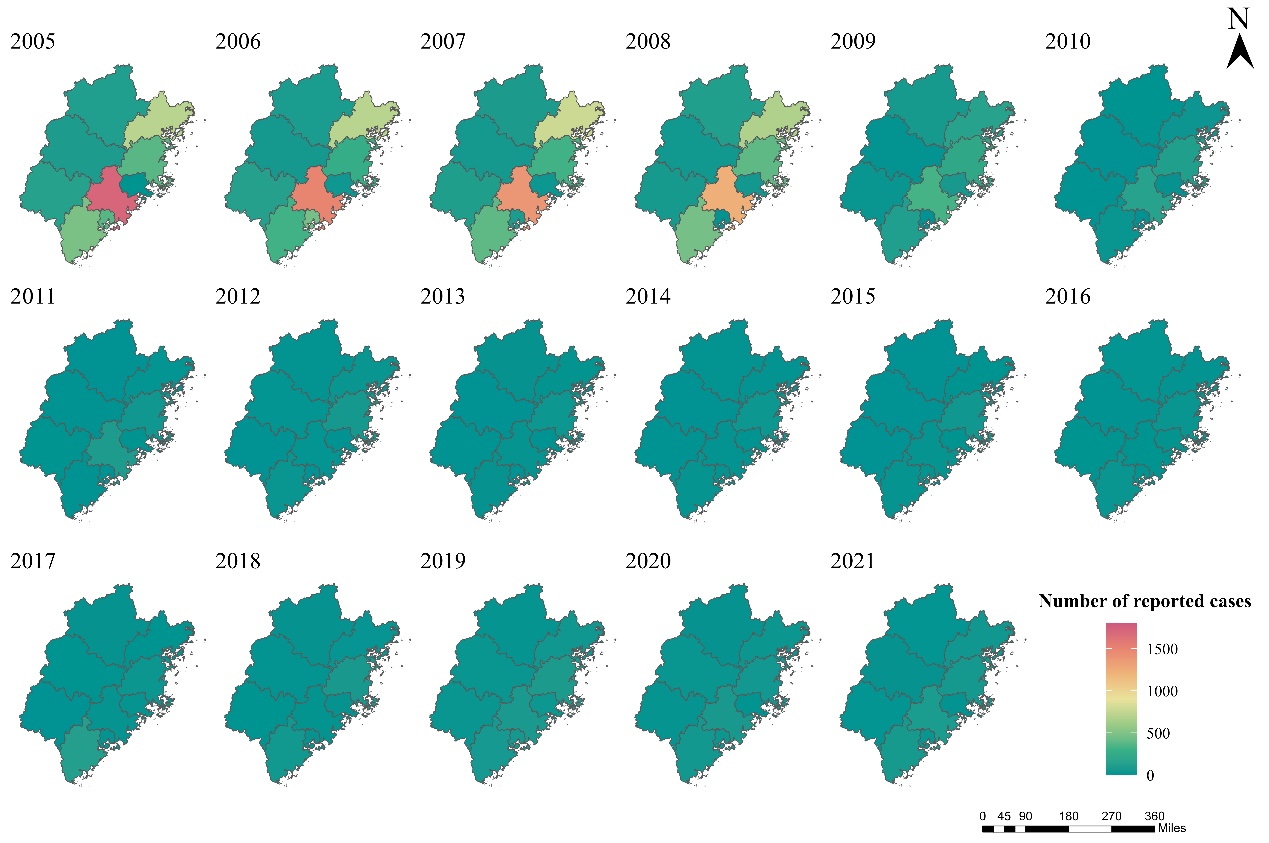


**Figure S5.** The geographical distribution of annualized average incidence of No pathogenic findings reported PTB in Fujian Province from 2005 to 2021.


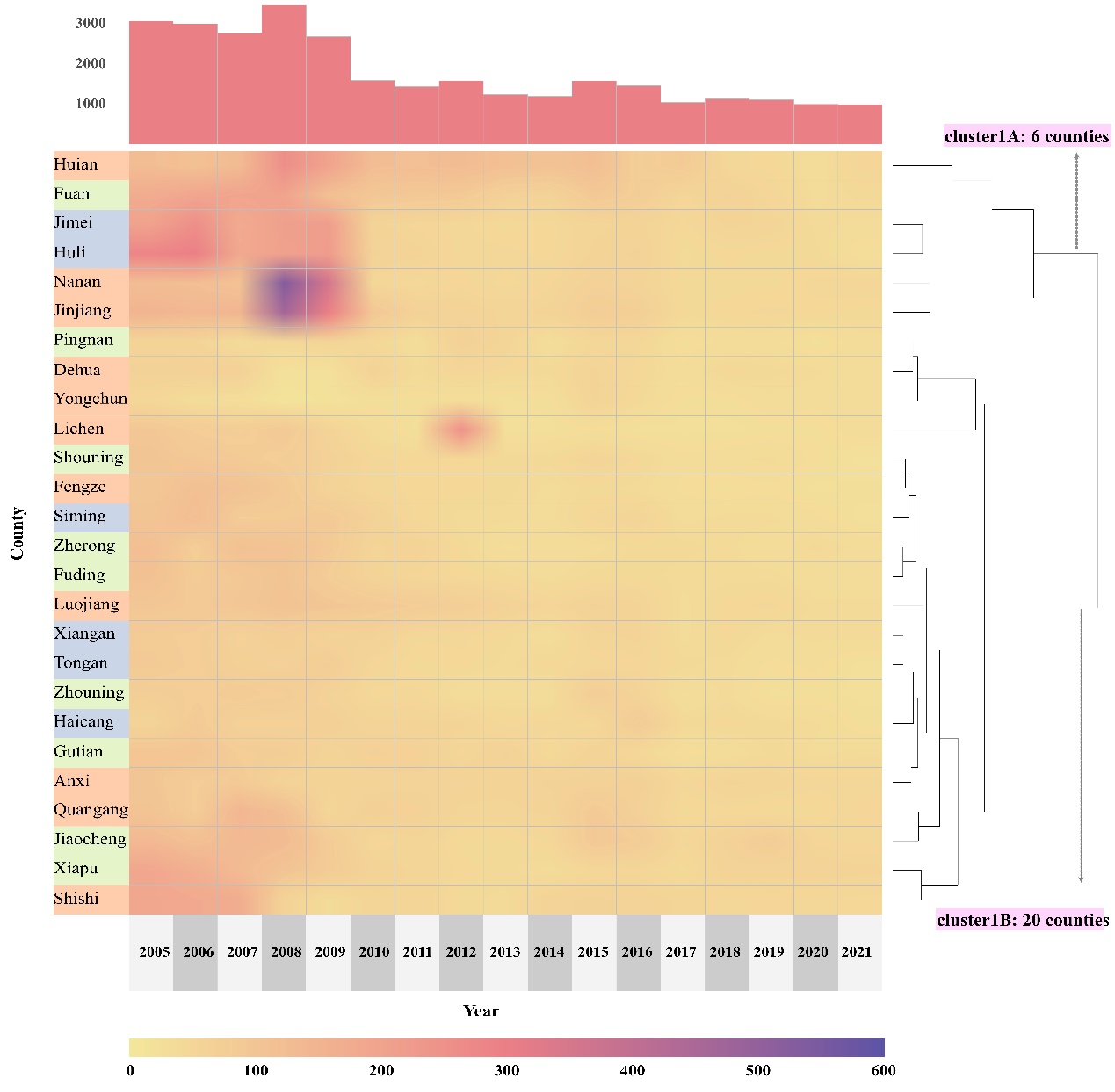


**Figure S6.** Hierarchical Clustering analysis of the regions in Fujian Province based on the incidence of PTB from 2005 to 2021. **This heatmap was based on the incidence of PTB in 26 county-level cities of prefecture-level cities in Cluster 1;**


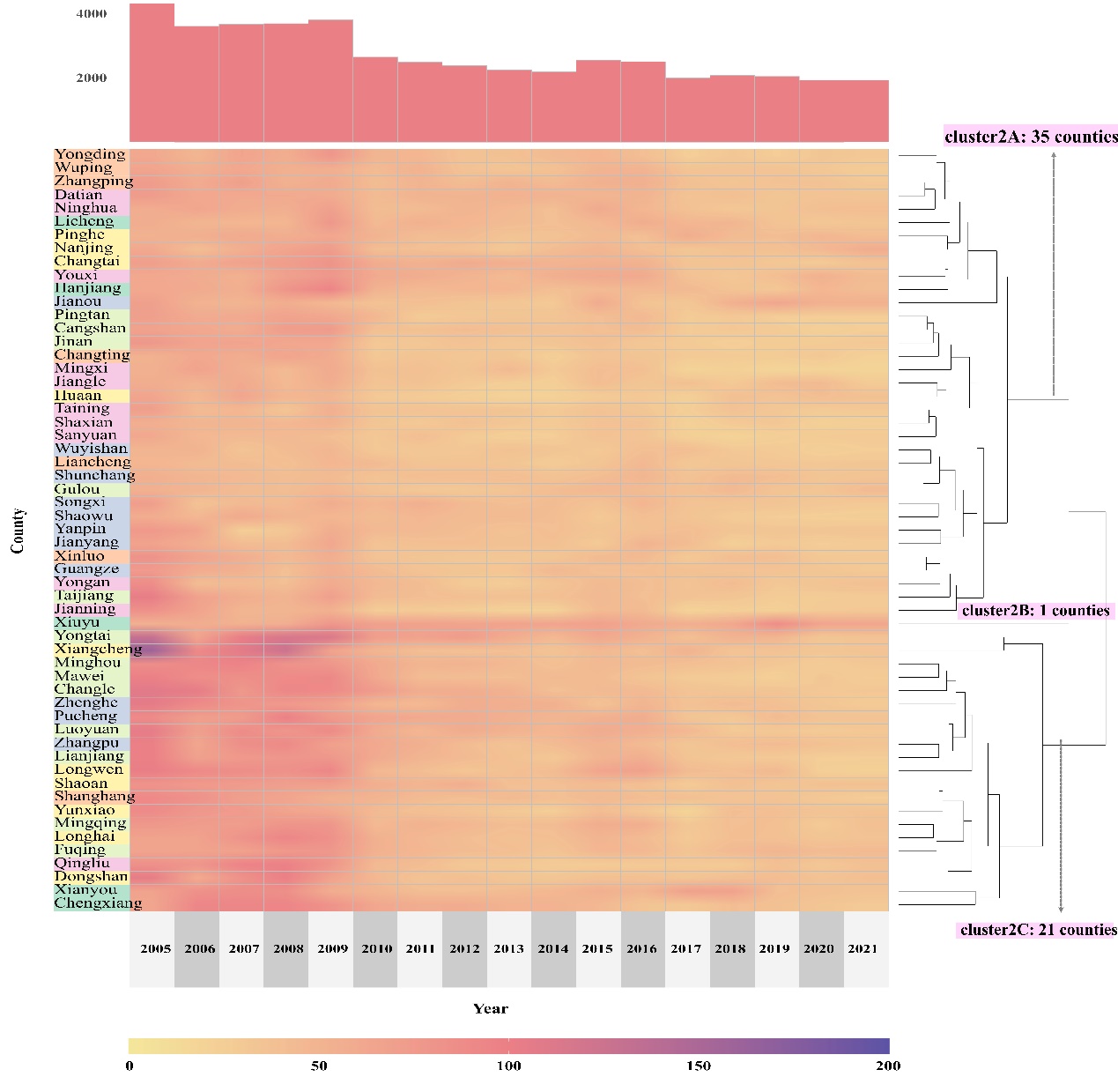


**Figure S7.** Hierarchical Clustering analysis of the regions in Fujian Province based on the incidence of PTB from 2005 to 2021. **This heatmap was based on the incidence of PTB in 57 county-level cities of prefecture-level cities in Cluster 2.**
