# Supplementary material for: Analysis of Tuberculosis Epidemiological Distribution Characteristics in Fujian Province, China, 2005-2021: Spatial-Temporal Analysis Study
Source: JMIR Public Health Surveill. 2024 Nov 18;10:e49123. doi: 10.2196/49123 (PMC11590169; doi:10.2196/49123)
Supplement: Multimedia Appendix 2 [file publichealth-v10-e49123-s002.docx]

Three methods were used to analysis TB epidemiology characteristics.

Firstly, Join-point regression models were used to calculate the TB temporal trends, which is often used to describe changes in trends. The annual percentage change (APC) for tuberculosis incidence rates was used to quantify the time trends (Equation 1-3), and it also estimates the average annual percentage change (AAPC) in the whole period studied[26, 27]. APC assumes that the annual mortality change is continuously stable throughout the observed period, according to the same percentage as the previous year for log-linear change to describe the trend of mortality over time, which is a common indicator for the evaluation of the temporal trend of mortality, calculated as follows,

$APC=\left\{ e^{\beta}-1 \right\}*100$ (1)

$Lower=100*\left\{ e^{\left( \beta-s*t_{d}^{-1}\left( 1-\alpha/2 \right) \right)-1} \right\}$ (2)

$Upper=100*\left\{ e^{\left( \beta+s*t_{d}^{-1}\left( 1-\alpha/2 \right) \right)-1} \right\}$ (3)

Where *β* is the regression coefficient; APC of 100*(1- α) % confidence interval is calculated as above, where *d* is the degree of freedom, *s* is the standard error of the regression coefficient *β*, and $t_{d}^{-1}\left( q \right)$is the *q* percentile of the *t* distribution with degree of freedom *d*.

Secondly, spatial autocorrelation index is an important index to measure the potential interconnectedness and dependence of study objects in a region. The Moran index (Moran's I) is a statistical method used to measure spatial autocorrelation, which can help analyze clustered or discrete patterns in geographic data or other data sets with spatial properties. It is divided into Global Moran’s I (*I*) and Local Moran’s I (*I_i_*). The values of Global Moran’s I (Equation 4) are in the range of [−1, 1]. At a given significance level (generally 5%), the value > 0 indicates a positive spatial correlation, and a larger value indicates a more significant spatial correlation; the value < 0 indicates a negative spatial correlation, and a smaller value indicates a greater spatial difference, while the value = 0 indicates a random spatial distribution.

Local Moran’s I (Equation 5) subdivide spatial correlation patterns into four types, specifically, H-H and L-L of a positive spatial correlation (that is, spatial units with high or below-average attribute values surrounded by domains with high or below-average attribute values), and H-L and L-H of a negative spatial correlation (that is, spatial units with high or below-average attribute values surrounded by domains with low or above-average attribute values).

Although Moran’s I index can reflect the clustering characteristics of TB epidemic, it cannot determine whether the spatially aggregated area is aggregated by hot spot values or cold spot values. Therefore, the GetisOrd Gi* statistic (Equation 6) is used to identify the statistically significant hot and cold spots. The Gi∗ statistic is a z-score, so no further calculations are required for a statistically significant positive z-score, the higher the z-score, the tighter the clustering of high values (hot spots). For a statistically significant negative z-score, the lower the z-score, the tighter the clustering of low values (cold spots).

They are calculated based on the equation as follows, where, *n* represents the number of study objects, *x_i_* and *x_j_* are observed values of study units *i* and *j*, $\bar{x}$is the average of *x_i_*, *W_ij_* is a spatial weight matrix, and *s* is the standard deviation of the incidence rate over the overall region. In this paper, ARCGIS 10.8 is used for the spatial autocorrelation analysis, where the spatial weight matrix is a distance-based spatial weight matrix, and the parameters are all default values of the software. The distance is measured by the “Contiguity-Edges Corners”.

$I=\frac{n\sum_{i=1}^{n} \sum_{j=1}^{n} W_{ij}\left( x_{i}-\bar{x} \right)\left( x_{j}-\bar{x} \right)}{\left( \sum_{i=1}^{n} \sum_{j=1}^{n} W_{ij} \right)\sum_{i=1}^{n} \left( x_{j}-\bar{x} \right)^{2}}$,$\bar{x}=\frac{1}{n}\sum_{i=1}^{n} x_{i}$,i≠j (4)

$I_{i}=\frac{n^{2}}{\sum_{i=1}^{n} \sum_{j=1}^{n} W_{ij}}*\frac{\left( x_{i}-\bar{x} \right)\sum_{j=1}^{n} W_{ij}\left( x_{i}-\bar{x} \right)}{\sum_{j=1}^{n} \left( x_{i}-\bar{x} \right)^{2}}$ (5)

$G_{i}^{*}=\frac{\sum_{j=1}^{n} W_{ij}x_{ij}-W_{i}^{*}x}{s\sqrt{\left( nS_{1i}-\left( W_{i}^{*} \right)^{2}/\left( n-1 \right) \right)}}$,$W_{i}^{*}=\sum_{j=1}^{n} W_{ij}$,$S_{1i}=\sum_{j=1}^{n} {W_{ij}}^{2}$(6)

Lastly, cluster analysis is used to further explore the internal relationships of different clusters. Unsupervised hierarchical cluster analysis was conducted to identify relevant groups of tuberculosis patients based on 9 prefecture-level cities and 83 county-level cities. The analysis was performed using the *R* statistical software package with a Euclidean distance measure and Ward linkage. In addition, statistical analyses for association and correlation were conducted using a variety of methods including Fisher's exact test, analysis of variance, chi-square test and the independent sample t-test to further investigate the relationship between the identified clusters and relevant demographic and clinical characteristics of the patient population.
